# Giving Animal Food to Infants

**Published:** 1891-05

**Authors:** 


### GIVING ANIMAL FOOD TO INFANTS.

There is no greater error in the management of children than that of giving them animal diet very early. To feed an infant with solid animal food before it has teeth proper for masticating, shows a total disregard of the plain indications of Nature in withholding teeth suited to this purpose until the age at which the system requires solid food. Before that time, milk, farinaceous food, and animal broths, afford the kind of sustenance which is at once best suited to the digestive organs and to the nutrition of the system. The method of mincing and pounding meat as a substitute for mastication may do very well for the toothless octogenarian whose stomach has been habituated to concentrated nutriment, but the digestive organs of a child are not adapted to the due preparation of such food, and will be disordered by it.

